# Generation and characterization of a Müller-glial-cell-specific *Il6ra* knockout mouse to delineate the effects of IL-6 trans-signaling in the retina

**DOI:** 10.1038/s41598-022-22329-3

**Published:** 2022-10-21

**Authors:** Rebekah Robinson, Joshua Glass, Ashok Sharma, Shruti Sharma

**Affiliations:** 1 Center for Biotechnology and Genomic Medicine, Medical College of Georgia, Augusta University, 1120 15th Street, CAII 4139, Augusta, GA 30912 USA; 2 Department of Population Health Sciences, Augusta University, Augusta, GA USA; 3 Culver Vision Discovery Institute, Augusta University, Augusta, GA USA; 4 Department of Ophthalmology, Augusta University, Augusta, GA USA

**Keywords:** Extracellular signalling molecules, Interleukins

## Abstract

Interleukin-6 (IL-6) is implicated in various retinal and vascular complications associated with diabetic retinopathy (DR). This cytokine functions through two main modalities: classical signaling, in cells expressing the membrane-bound receptor (IL-6Rα); and trans-signaling, possible in most cells through a soluble form of the receptor (sIL-6R). These pathways are considered to be anti-inflammatory and pro-inflammatory, respectively. Our recent studies in retinal endothelial cells and diabetic mice have shown that inhibiting only IL-6 trans-signaling is sufficient to prevent increased vascular leakage, oxidative stress, and inflammation characteristic of DR. Isolating the specific effects of each signaling pathway, however, remains difficult in cells expressing IL-6Rα that are thus capable of both classical and trans-signaling. Müller glial cells (MGCs), the most abundant retinal macroglial cells, span the entire retinal thickness with vital roles in maintaining retinal homeostasis and regulating the blood-retinal barrier through secreted factors. The specific effects of IL-6 trans-signaling in MGCs remain poorly understood given their responsiveness to both IL-6 signaling modalities. In this study, we addressed these concerns by generating an MGC-specific knockout mouse using Cre-*loxP* deletion of the *Il6ra* cytokine-binding region. We assessed transcriptional and translational *Il6ra* expression to confirm the knockout and characterized the effects of knockout on visual functioning in these mice*.*

## Introduction

The pleiotropic cytokine interleukin-6 (IL-6) is increasingly emerging as an important contributor to the pathology of diabetic retinopathy (DR). Numerous studies have shown that serum IL-6 levels are elevated in DR patients, and ocular IL-6 levels are positively correlated with worsening retinal pathology and aberrant vascular proliferation^1–6^. IL-6 also has neuroprotective functions in the retina and enhances survival of retinal ganglion cells^7,8^. We and others have suggested that these conflicting functions of IL-6 in the retina are possible through the cytokine’s two distinct signaling mechanisms using either a membrane-bound (classical signaling) or soluble receptor (trans-signaling)^5,9–14^. Both mechanisms activate signal transducer and activator of transcription 3 (STAT3), while trans-signaling has been shown to also activate phosphoinositide 3-kinase (PI3K) and mitogen activated protein kinase (MAPK)^15–18^. It has also been hypothesized that the availability of the various receptors and signaling components can allow for cell-type specific differences in signaling response, further complicating the study of IL-6 in highly specialized tissues like the retina.

Our recent studies in human retinal endothelial cells and streptozotocin (STZ) induced diabetic mice indicate that the adverse effects of IL-6 in DR are primarily mediated by IL-6 trans-signaling^5,13^. Diabetic mice treated with the selective trans-signaling inhibitor, sgp130Fc, showed significantly less oxidative damage than untreated diabetic controls. This reduction in oxidative damage was seen across all layers of the retina, suggesting that IL-6 trans-signaling may contribute to damage in retinal cell types in addition to the retinal vasculature.

Although several retinal cell types are affected in DR, the role of retinal Müller glial cells (MGCs) in the pathobiology of DR remains elusive. MGCs are the predominant glial cell in the retina, comprising ~ 90% of glial cells in the eye and spanning the entire width of the retina. MGCs also help in maintaining the blood-retinal barrier (BRB) by promoting barrier properties in retinal endothelial cells^19–31^, and conditional ablation of Müller glia leads to BRB breakdown^32^.

Studies evaluating IL-6 trans-signaling in MGCs are complicated by the fact that MGCs express the IL-6 receptor (IL-6Rα) and are thus able to respond to IL-6 through both the classical and trans-signaling pathways, making it difficult to delineate the individual effects of each signaling mechanism. Current inhibitors of IL-6 (siltuximab) or IL-6R (tocilizumab) globally inhibit both classical and trans-signaling, and, though a selective inhibitor of IL-6 trans-signaling has been developed (sgp130Fc)^33^, no selective classical signaling inhibitor is currently available. IL-6 trans-signaling can be selectively activated in cells using exogenous IL-6 and soluble IL-6R (sIL-6R), with sIL-6R in excess^7,13^, or using the designer cytokine, Hyper IL-6, which consists of an IL-6 and sIL-6R complex connected through a peptide linker^34^. However, both strategies are not feasible for studies using animal models, especially when the goal is to evaluate the specific effects of trans-signaling in MGCs that naturally express IL-6Rα. With expression of IL-6Rα and the presence of endogenous IL-6, the activation of IL-6 classical signaling cannot be excluded.

To inhibit classical signaling and allow only trans-signaling in Müller glial cells in vivo, we have successfully generated a Müller-glial*-*cell-specific *Il6ra* knockout mouse by crossing homozygous *Il6ra*-floxed mice with Müller-glial-cell-specific *Pdgfra-Cre* transgenic mice. This mouse was generated using strains expressing an MGC-specific Cre recombinase^35,36^ and floxed *Il6ra*, with two *loxP* sites flanking exons 4–6^37^. Crossing these strains results in the deletion of much of the cytokine-binding domain of the *Il6ra* gene and produces a non-functional IL-6Rα in MGCs.

## Methods

### Mice

Three mouse strains were purchased from the Jackson Laboratory: wildtype (C57BL/6J) mice, male homozygous floxed *Il6ra* (B6;SJL-*Il6ra*^*tm1.1Drew*^/J) mice, and female mice expressing a Müller-glial-cell-specific Cre recombinase (C57BL/6-Tg(Pdgfra-cre)1Clc/J). Male floxed *Il6ra* mice and female MGC-specific *Pdgfra-Cre* mice were crossed, and heterozygous female offspring expressing Cre were backcrossed to parental floxed *Il6ra* males to generate a colony of MGC-specific *Il6ra*^*−/−*^ (KO) mice. Littermate controls were housed separately for comparison with the KO, including homozygous *Il6ra*^*fl/fl*^ Cre-negative, heterozygous *Il6ra*^*wt/fl*^ Cre-positive, and *Il6ra*^*wt/wt*^ Cre-positive mice. Knockout mice characterized in this study were maintained in a heterozygous state for Cre to exclude any possible confounding dosage effect. This study was conducted in accordance with the ARVO Statement for the Use of Animals in Ophthalmic and Vision Research and the Animal Research: Reporting of In Vivo Experiments (ARRIVE) guidelines. All resources and efforts were used to ensure the safe and humane treatment of animals used in the study to minimize any possible suffering during experimental procedures and euthanasia. The animal protocol (Protocol #2014-0676) was approved by the Institutional Animal Care and Use Committee (IACUC) at Augusta University.

### Genotyping

The genotypes of parental *Il6ra*^*fl/fl*^ and *Pdgfra-Cre* mice, as well as their offspring, were determined using PCR to evaluate the zygosity of floxed *Il6ra* alleles and the expression of Cre in genomic DNA isolated from ear punch tissue as previously described^38^. Briefly, the tissue was lysed in 0.1 M NaOH with 0.2 mM EDTA for 1 h at 95 °C. The solution was neutralized with 40 mM Tris (pH 5.0), and residual tissue was pelleted by centrifugation. PCR was performed using SsoAdvanced Universal SYBR Green Supermix and Bio-Rad CFX Connect Thermocycler (Bio-Rad Laboratories, Hercules, CA), and allele-specific primers were purchased from Integrated DNA Technology (San Jose, CA, USA). PCR product length for each reaction was verified by 1.5% agarose gel electrophoresis using a GeneRuler 1 kb Plus DNA Ladder (SM1332, Thermo Scientific, Waltham, MA, USA), with bands visualized using a ChemiDoc Imaging System (Bio-Rad). The wildtype and floxed *Il6ra* alleles were detected by PCR amplification of 109 bp and 123 bp fragments, respectively, while Cre expression was determined by PCR amplification of a 635 bp fragment. Floxed mice (*Il6ra*^*fl/fl*^) were distinguished from heterozygous floxed (*Il6ra*^*wt/fl*^) littermates by *Il6ra*^*fl/fl*^ lacking a WT band on the gel. Genotyping primer sequences for *Il6ra* were provided by The Jackson Laboratory as summarized in Supplemental Table 1^39^.

### Isolation of Müller glial cells (MGCs)

MGCs were isolated from WT and KO mice at P7 using techniques modified from previously published protocols^40–43^. Briefly, mouse pups were euthanized with isoflurane followed by decapitation, and whole eyes were gently removed and placed in 5 mL Dulbecco’s Modified Eagle Medium (DMEM: 1 g/L glucose, 25 mM HEPES, 1 mM sodium pyruvate, 4 mM l-glutamine; Gibco, Thermo Fisher Scientific, Waltham, MA, USA) supplemented with 0.1% penicillin/streptomycin (Cytiva, Thermo Fisher Scientific, Waltham, MA, USA) at room temperature overnight. Eyes from each pup were stored separately to maintain individual MGC populations. The next day, eyes were washed with 1× Dulbecco’s Phosphate Buffered Saline (DPBS) warmed to 37 °C and digested with 0.25% trypsin/EDTA (Corning; Corning, NY, USA) containing 2000 units/mL collagenase IV (Cat. # LS004186, Worthington Biochemicals, Lakewood, NJ, USA) for 1 h at 37 °C in a humidified incubator containing 5% CO_2_. Under a dissecting microscope, retinal tissue was separated from the cornea, sclera, lens, and retinal pigment epithelium and then carefully transferred to a dish containing DMEM supplemented with 10% FBS and 1% penicillin/streptomycin. For each mouse, retinal tissue from both eyes was pooled and incubated in a 10-cm cell culture dish at 37 °C in a humidified incubator containing 5% CO_2_ for 7–10 days, until isolated Müller glial cells reached confluence. Cells were split and passaged 1–2 times, until there were no retinal pigment epithelial cells remaining in the culture, as identified by their brown-colored pigment. MGC purity was verified by immunofluorescence detection of vimentin (Fig. 3B).

### RT-PCR analysis

RNA was isolated from MGCs using phenol–chloroform extraction with TRIzol reagent (Invitrogen, Carlsbad, CA, USA) as previously described^44^. Briefly, cells were lysed in TRIzol, and RNA was extracted with chloroform and centrifugation for 15 min at 12,000×*g* at 4 °C. The aqueous phase containing RNA was carefully transferred into a new RNAse-free tube, precipitated with isopropanol, and washed four times with 75% ethanol. The RNA pellet was air dried for 10 min, resuspended in nuclease-free water, and heated at 55 °C for 10 min. cDNA was synthesized using High-Capacity cDNA Reverse Transcriptase Kit (Applied Biosystems, Foster City, CA, USA) with 1 µg RNA per sample following the manufacturer’s protocol. All primers for RT-PCR were designed using the NCBI Primer-BLAST tool, purchased from Integrated DNA Technology (Supplemental Table 2), and RT-PCR reactions were performed using SsoAdvanced Universal SYBR Green Supermix and Bio-Rad CFX Connect Thermocycler (Bio-Rad Laboratories, Hercules, CA, USA). Primer sequences were selected to determine *Il6ra* expression at the exon 5–6 region (E5–E6) of the gene flanked by *loxP* sites and containing the cytokine-binding segment of *Il6ra*. Two other primer sets were used to compare mRNA expression in regions outside the *loxP*-flanked segment for the exon 2–3 region (E2–E3) and the exon 10 (E10) region. Similarly, *Cre* expression was measured at the mRNA level in isolated MGCs using RT-PCR to confirm its presence in KO and its absence in WT. To assess glial cell activation, *Gfap* expression was also compared between WT and KO MGCs. Gene expression was normalized to the expression of *Gapdh*, and relative mRNA expression values were calculated as 2^−ΔΔCq^ and represented as fold control relative to WT.

### Western blot analysis

MGC lysates and tissue homogenates were prepared in radioimmunoprecipitation (RIPA) buffer (Cell Biolabs, Inc., San Diego, CA, USA) containing protease and phosphatase inhibitors (Sigma-Aldrich). Equal amounts of protein (20–40 µg per sample) were denatured in Laemmli buffer (Thermo Scientific), heated to 100 °C for 10 min, and then loaded in a pre-cast 4–15% sodium dodecyl sulfate–polyacrylamide gel (SDS-PAGE) (Bio-Rad) for electrophoresis. Proteins were separated at 100–120 V and transferred to a nitrocellulose membrane, and the membrane was blocked in 5% milk in TBST for 2–3 h at room temperature. After blocking, the membrane was incubated with anti-IL6Rα antibody (MA5-29721, Invitrogen, 1:500) at 4 °C overnight. The membrane was washed 3 times in TBST and then incubated with horseradish peroxidase (HRP)-conjugated anti-rabbit secondary antibody (31460, Invitrogen) for ~ 2 h at room temperature. The membrane was washed and then developed with ECL substrate (Thermo Scientific), and images were captured with the ChemiDoc Imaging System (Bio-Rad). Bands were quantified using ImageJ software (NIH), and IL-6Rα expression values were normalized to the expression of β-actin (AC028, ABclonal). Quantified western blot data was presented as fold control relative to normalized wildtype expression.

### Immunofluorescence staining

Isolated MGCs were plated on gelatin-coated imaging plates (Eppendorf; Enfield, CT) and grown to confluency. Cells were washed with DPBS, fixed with ice-cold 80% acetone for 10 min at RT, and permeabilized with 0.25% Triton X-100 for 10 min at RT prior to blocking in 10% normal goat serum (Life Technologies) for 2–4 h at RT. Cells were then incubated with primary antibodies at 4 °C overnight: anti-IL6Rα antibody (sc-373708, Santa Cruz, 1:100), anti-vimentin antibody (5741S, Cell Signaling, 1:100), or anti-GFAP antibody (A0237, ABclonal, 1:100). For double labeling of IL-6Rα and vimentin, MGCs were incubated with both primary antibodies simultaneously. Cells were washed three times with DPBS and then incubated with respective secondary antibodies conjugated to Alexa Fluor 488 (Donkey anti-Rabbit, A21206, Invitrogen, Carlsbad, CA, USA) or Alexa Fluor 546 (Donkey anti-Mouse, A10036, Invitrogen) for 1 h at RT. Cells were washed three times and counterstained with 4′,6-diamidino-2-phenylindole (DAPI) for 10 min (1:2000, Thermo Scientific). Images were acquired using a Leica STELLARIS Confocal Microscope (Buffalo Grove, IL, USA) at 40× magnification.

### Immunohistochemistry

For immunostaining of retinal sections, whole eyes were isolated following euthanasia, immediately embedded in Tissue-Tek OCT compound (Sakura Finetek USA, Torrance, CA, USA), and stored at − 80 °C until further analyses. Retinal sections were stained with hematoxylin and eosin (H&E) to visualize retinal thickness and morphology. For immunohistochemistry analysis, cryosections were incubated overnight with anti-IL6Rα antibody (sc-373708, Santa Cruz, 1:100) and anti-EEAT1/SLC1A3 (GLAST) antibody (A15722, ABclonal, 1:100), followed by the appropriate secondary antibody conjugated to Alexa Fluor 488 or Alexa Fluor 546 (Invitrogen, Carlsbad, CA, USA) for 1 h at RT. Nuclei were counterstained with DAPI, and fluorescent images were captured with a Leica STELLARIS Confocal Microscope at 20× or 63× magnifications.

### Allele-specific PCR analysis of ***Pde6b***^***rd1***^, ***Crb1***^***rd8***^, and ***Gpr179***^***nob5***^ mutations

The presence of wildtype alleles of *Pde6b*, *Crb1*, and *Gpr179* genes or their *rd1*, *rd8*, and *nob5* mutant alleles, respectively, was evaluated in wildtype (C57BL/6J), B6;SJL-*Il6ra*^*tm1.1Drew*^/J, C57BL/6-Tg(Pdgfra-cre)1Clc/J, and MGC-specific *Il6ra*^*−/−*^ mice using allele-specific PCR of genomic DNA as previously described^45,50–54^. PCR reactions were performed with SsoAdvanced Universal SYBR Green Supermix and Bio-Rad CFX Connect Thermocycler (Bio-Rad Laboratories), and PCR product length was determined via 1.5% agarose gel electrophoresis using a GeneRuler 1 kb Plus DNA Ladder (SM1332, Thermo Scientific, Waltham, MA) and ChemiDoc Imaging System for gel visualization (Bio-Rad). Primer sequences used for PCR amplification are summarized in Supplemental Table 1.

### Visual function testing

For live retinal testing, mice were anesthetized with ketamine (80 mg/kg) and xylazine (16 mg/kg), and pupils were dilated with 1% tropicamide ophthalmic solution (Akorn, Inc.). Retinal images were acquired using a Phoenix MICRON IV imaging system in bright field mode, which was immediately followed by a 10 µL subcutaneous injection of fluorescein dye (AK-FLUOR 10%, 100 mg/mL) for assessment of the retinal vasculature by fluorescein angiography. For optical coherence tomography (OCT), retinal thickness was measured in anesthetized mice by using a Bioptigen Spectral Domain Ophthalmic Imaging System (SDOIS; Bioptigen Envisu-R2200). Briefly, 1% tropicamide was used to dilate pupils, and the eyes were lubricated with GenTeal Tears PF Lubricant Moderate Eye Drops (Alcon Laboratories, Inc.) prior to acquiring OCT measurements from each eye. Data was analyzed using the InVivoVueTM Diver 2.4 software (Bioptigen, Inc., Durham, NC, USA). For electroretinography (ERG), mice were dark-adapted overnight, and all readings were acquired in a dark room without background lights. The pupils of anesthetized mice were dilated, and mice were placed on the heated platform of a Phoenix MICRON™ Ganzfeld ERG system. Eyes were coated with a drop of hypromellose ophthalmic lubricant (Goniovisc), and reference and ground leads were placed under the skin with platinum needle electrodes in the forehead and tail, respectively. Readings were measured with the gold-plated corneal contact electrode aligned to the pupil using the internal camera with flashes of green light (-1.7, -0.8, 0.1, and 2.8 log(cd s/m^2^)) followed by UV light (-0.8, 0.1, 1.0, and 2.8 log(cd s/m^2^)). Measurements were recorded for both right and left eyes, and A-wave and B-wave amplitudes at each flash intensity were calculated by the Ganzfeld ERG software using an average of 3–10 successive flash readings at each intensity.

### Statistical analyses

Statistical significance for data in this study was defined as p-value < 0.05 (knockout vs. wildtype) and calculated using GraphPad Prism software 9.0 (GraphPad Software, Inc., San Diego, CA, USA). Statistical analyses of RT-PCR data for *Il6ra* were performed by a Two-way ANOVA with Tukey’s multiple comparisons, while no significance was determined for *Cre* expression due to a lack of detection in wildtype. An unpaired T-test was used to assess the significance of *Gfap* mRNA expression from RT-PCR analysis. Unpaired T-tests were also used to determine the significance of quantified data from western blot, OCT, and ERG analyses, comparing changes in MGC-specific *Il6ra*^*−/−*^ to wildtype or littermate controls.

## Results

### Generation of Müller-glial-cell-specific *Il6ra* knockout mice

**Figure 1:**
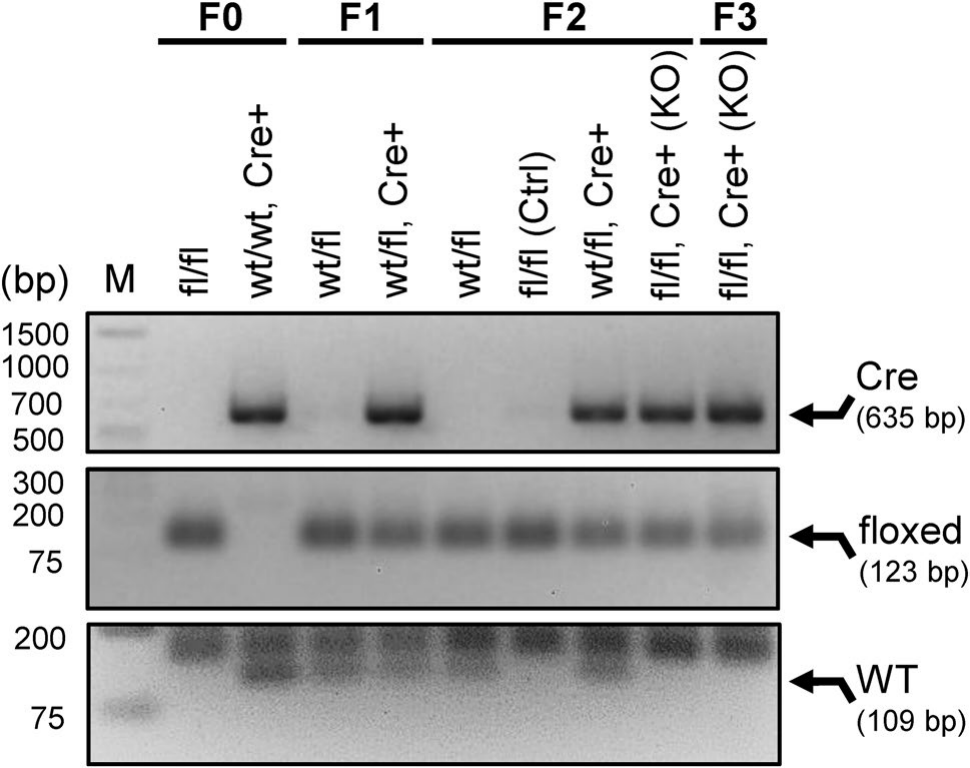
Genotyping schematic used for the generation of Müller-glial-cell-specific *Il6ra*^*−/−*^ mice. A representative agarose gel shows genotyping PCR products for *Cre* (635 bp) and floxed (123 bp) or wildtype (WT, 109 bp) *Il6ra* alleles in mice from F0–F3 generations. Genotyping results are shown for F0 (fl/fl and wt/wt Cre+), F1 (wt/fl and wt/fl Cre+), F2 (wt/fl, fl/fl, wt/fl Cre+, and fl/fl Cre + KO), and F3 (fl/fl Cre + KO); M, GeneRuler 1 kb Plus DNA Ladder.

The genotypes of all mice used in this study were determined by allele-specific PCR and agarose gel electrophoresis of PCR products for *Cre* (635 bp) and *Il6ra* wildtype (WT, 109 bp) or floxed (123 bp) alleles, as represented by 1.5% agarose gels in Fig. 1. Briefly, *Cre* amplification in F0 mice was verified in the C57BL/6-Tg(Pdgfra-cre)1Clc/J females (*wt/wt*, *Cre* +) with a single 635 bp length band that was absent in the B6;SJL-*Il6ra*^*tm1.1Drew*^/J (*fl/fl*) males *(fl/fl*). Floxed *Il6ra* was observed as a single band at 123 bp length for F0 *fl/fl* males 0, whereas F0 females expressing *Cre* only showed a band for the *Il6ra* WT allele at 109 bp length. These mice were crossed to produce an F1 generation heterozygous (*wt/fl*) for floxed *Il6ra*, with ~ 50% of these mice also being positive for *Cre* (*wt/fl*, *Cre* +). Genotyping of F1 mice confirmed all mice to have bands for both WT and floxed *Il6ra* alleles at 109 bp and 123 bp, respectively. Female F1 offspring positive for Cre recombinase DNA were backcrossed to the paternal floxed *Il6ra* strain to produce F2 offspring. Four genotypes were yielded from F2 breeding, including three genotypes present in F0 and F1 (*wt/fl*, *fl/fl*, and *wt/fl Cre* +) with the same band patterns. ~ 25% of remaining F2 mice were the first mice both positive for *Cre* and homozygous for floxed *Il6ra* (*fl/fl Cre* +) with the same band lengths on an agarose gel, thus yielding the first MGC-specific *Il6ra*^*−/−*^ (KO) in F2. Males and females of this genotype were subsequently used to breed F3 offspring, of which ~ 75% were both positive for *Cre* and homozygous for floxed *Il6ra* as F3 KO mice. Following successful genotyping, retinal tissues and MGCs were isolated from these F2 and F3 mice to confirm the efficacy and the specificity of the MGC-specific *Il6ra* knockout.

### Confirmation of *Il6ra* knockout in Müller glial cells in vitro and in vivo

**Figure 2:**
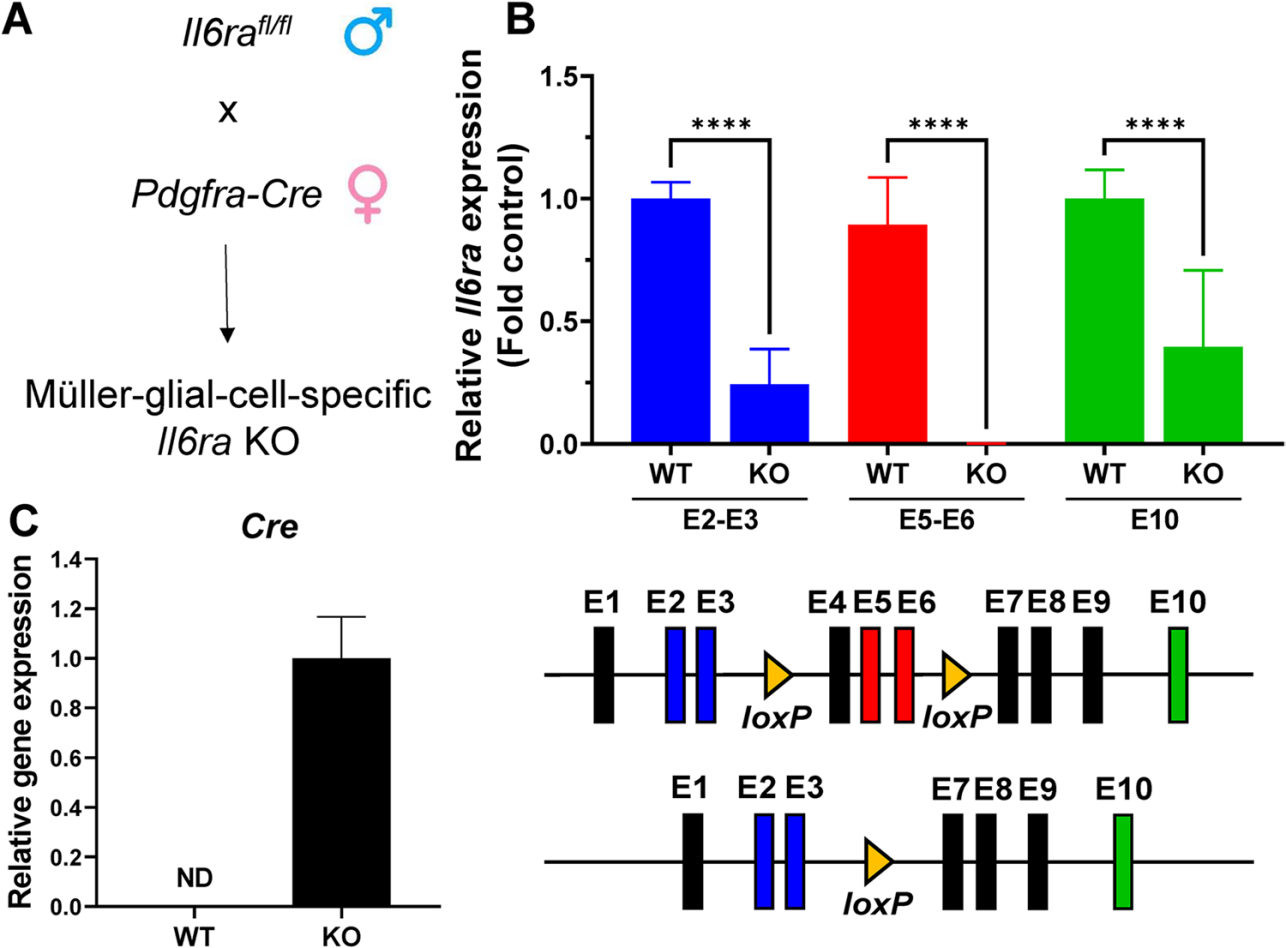
RT-PCR confirmation of *Il6ra*^*−/−*^ in isolated Müller glial cells (MGCs). (**A**) Overview of the breeding strategy used to generate MGC-specific *Il6ra*^*−/−*^ mice. (**B**) Relative *Il6ra* mRNA expression in isolated knockout MGCs (KO) compared to wildtype (WT) by RT-PCR using primers specific to the junction of exons 2 and 3 (E2–E3, blue), exons 5 and 6 (E5–E6, red), and within exon 10 (E10, green). Data is normalized to *Gapdh* expression (2^−ΔΔCq^) and reported as mean fold control (KO vs. WT) ± SD, n = 6–7/group, ****p-value < 0.0001 vs. WT (Two-way ANOVA, Tukey’s multiple comparisons test). (**C**) RT-PCR confirmation of *Cre* mRNA expression in KO MGCs normalized to *Gapdh*. As expected, *Cre* mRNA expression was not detected (ND) in WT MGCs. Data is represented as mean ± SD, n = 5/group; statistical significance was not calculated due to no detection in WT.

Müller glial cells were isolated from F2 and F3 knockout mice and cultured in vitro for comparison with cells isolated from wildtype mice. mRNA expression of *Il6ra* was measured using primers specific for three different regions across the gene—the junction of exons 2 and 3 (E2–E3), the junction of exons 5 and 6 (E5–E6), and within exon 10 (E10)^46^. Gene expression values were normalized to *Gapdh* expression as 2^−ΔΔCq^, and relative expression for each primer set was determined as fold change relative to wildtype. Interestingly, KO of *Il6ra* showed a significant decrease in expression for E2–E3 (FC = 0.244), E5–E6 (FC = 0.001), and E10 (FC = 0.396). While expression was detected with all three primer sets in wildtype MGCs, knockout cells showed nearly no detection of exon 5–6, which is within the region flanked by *loxP* sites in the *Il6ra* gene (Fig. 2A,B). This region contains the cytokine-binding domain of the IL-6 receptor, and an mRNA molecule lacking this region will not produce a functional protein^47^ (Supplemental Fig. 1). Additionally, we also confirmed the expression of Cre recombinase mRNA in knockout cells, which was absent in wildtype (Fig. 2C).

**Figure 3:**
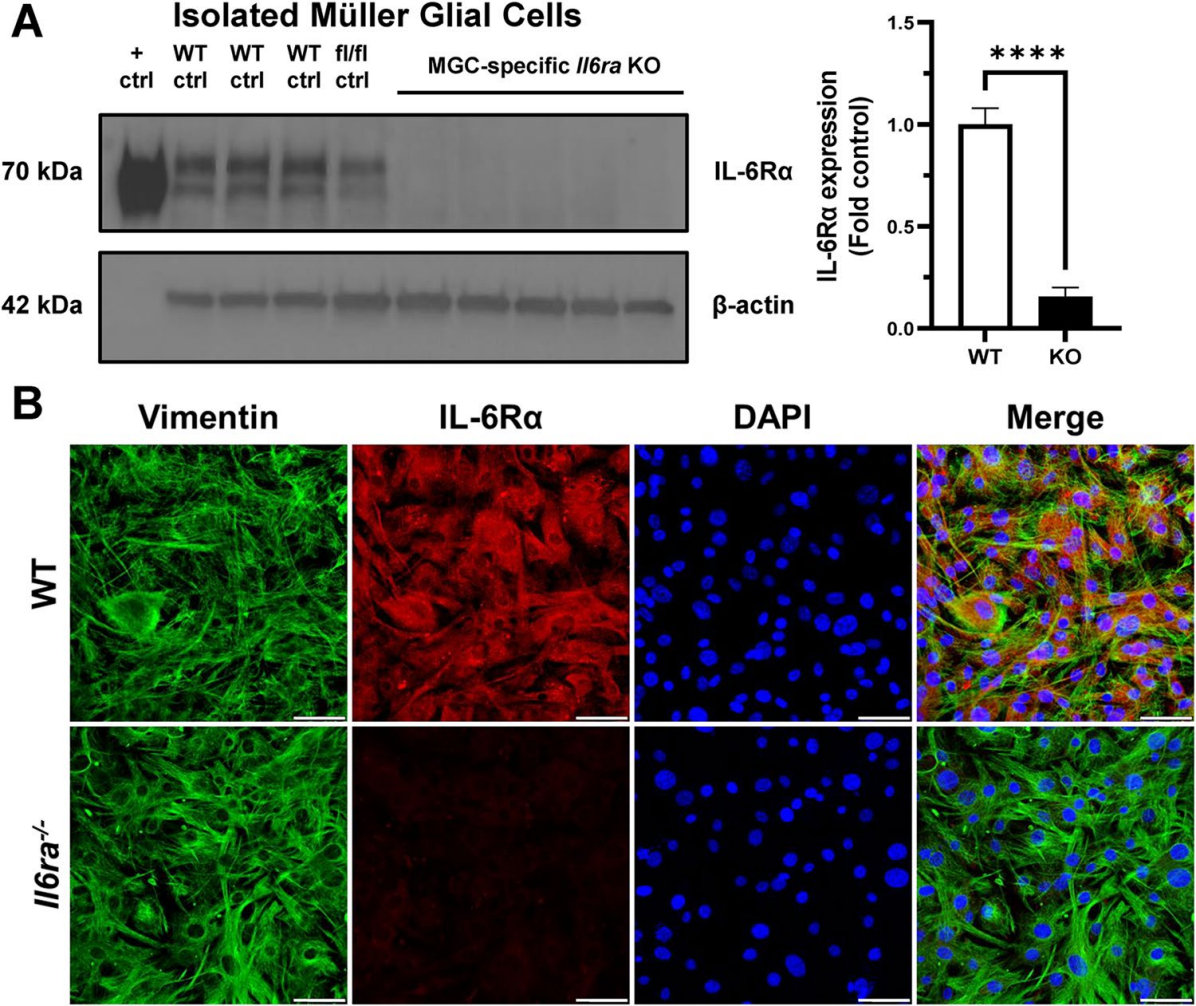
Western blot and immunofluorescence confirmation of *Il6ra*^*−/−*^ in isolated Müller glial cells (MGCs). (**A**) Knockout of *Il6ra* was assessed by western blot of MGCs isolated from wildtype (WT), floxed littermates (fl/fl), and MGC-specific *Il6ra*^*−/−*^ (KO) mice. Purified IL-6Rα protein (70 kDa) was used as a positive control (+ ctrl) for the membrane-bound receptor. Western blots were also probed for β-actin (42 kDa) for normalization. IL-6Rα protein expression was observed in WT (n = 3) and floxed (n = 1) MGC lysates but absent in KO MGCs (n = 5, FC = 0.156). Data is represented as fold control mean ± SD, n = 3–5/group, ****p-value < 0.0001 vs. WT (unpaired T-test). **(B)** Immunofluorescence staining for IL-6Rα (red) and vimentin (green) in isolated MGCs from WT and KO mice, counterstained with DAPI (blue). Images were captured using a Leica STELLARIS Confocal Microscope at 40× magnification (n = 4 technical replicates/group, n = 5–10 images/well).

Protein expression of membrane-bound IL-6Rα was detected by western blot in cell lysates from isolated MGCs to confirm the successful knockout of the receptor. Wildtype and *fl/fl* control samples showed normal IL-6Rα expression, but no expression was detected in the knockout cells (Fig. 3A). Similarly, IL-6Rα was also absent in immunofluorescence staining of isolated MGCs from knockout mice, while wildtype MGCs showed colocalized staining of the IL-6 receptor and vimentin (Fig. 3B). Both genotypes were positive for vimentin, a Müller glial cell marker used for determination of cell purity during isolation of MGCs, and have similar levels of GFAP expression (Supplemental Fig. 2). Thus, we confirmed that protein expression of IL-6Rα was successfully eliminated in vitro in MGCs isolated from these conditional *Il6ra*^*−/−*^ mice.

**Figure 4:**
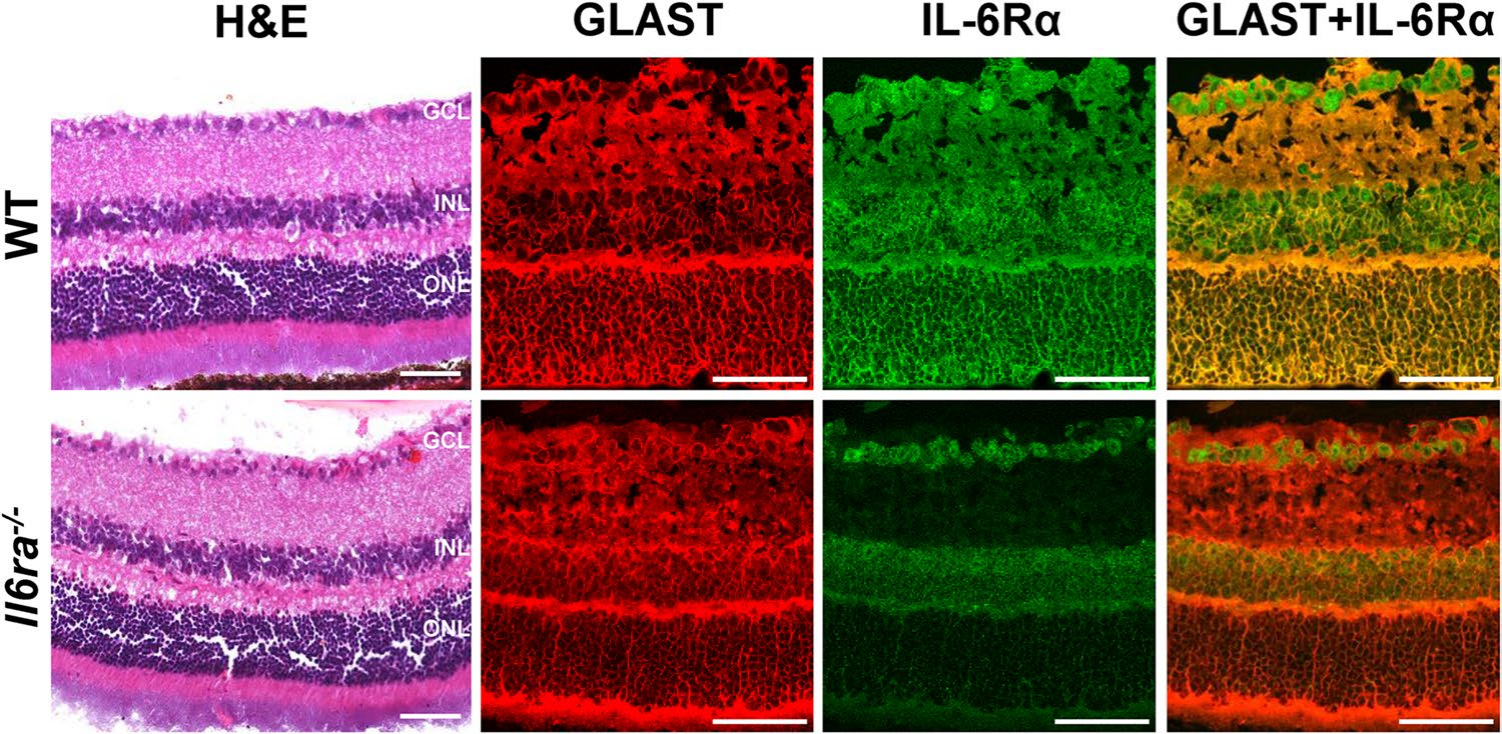
Immunofluorescence staining confirms a Müller-glial-cell-specific knockout of *Il6ra* in the retina. Representative images of MGC-specific *Il6ra* KO and wildtype (WT) mouse retinal sections stained with H&E and immunofluorescence for IL-6Rα (green) and GLAST (red), a marker of Müller glia, showing colocalization of GLAST and IL-6Rα (yellow). Data is representative of images acquired using a Keyence microscope (H&E) at 20× magnification and a Leica STELLARIS Confocal Microscope (immunofluorescence staining) at 63× magnification.

To assess retinal morphology and the cell specificity of *Il6ra* knockout in vivo, retinal sections from Müller cell-specific *Il6ra*^*−/−*^ mice and wildtype mice were stained with hematoxylin and eosin (H&E). Retinal morphology and thickness of the ganglion cell layer (GCL), inner nuclear layer (INL), and outer nuclear layer (ONL) were visibly unchanged by the knockout of *Il6ra* specific to MGCs (Fig. 4). Furthermore, immunofluorescence staining of retinal sections was performed using antibodies against IL-6Rα and glutamate/aspartate transporter (GLAST), a marker of Müller glia, to confirm the cell specificity of the knockout within the retina. The fluorescence intensity of IL-6Rα was significantly diminished throughout the KO retina compared to WT, and the colocalization of GLAST and IL-6Rα was abundant in the wildtype retina yet absent in the knockout retina (Fig. 4). Notably, the intensity of IL-6Rα in MGC-specific *Il6ra*^*−/−*^ retina was retained mostly within the GCL rather than the INL, the layer containing MGC nuclei, further supporting the specificity of the knockout only in MGCs and not in other retinal glial cells.

### Tissue IL-6Rα expression supports an *Il6ra* knockout specific to retinal Müller glia

**Figure 5:**
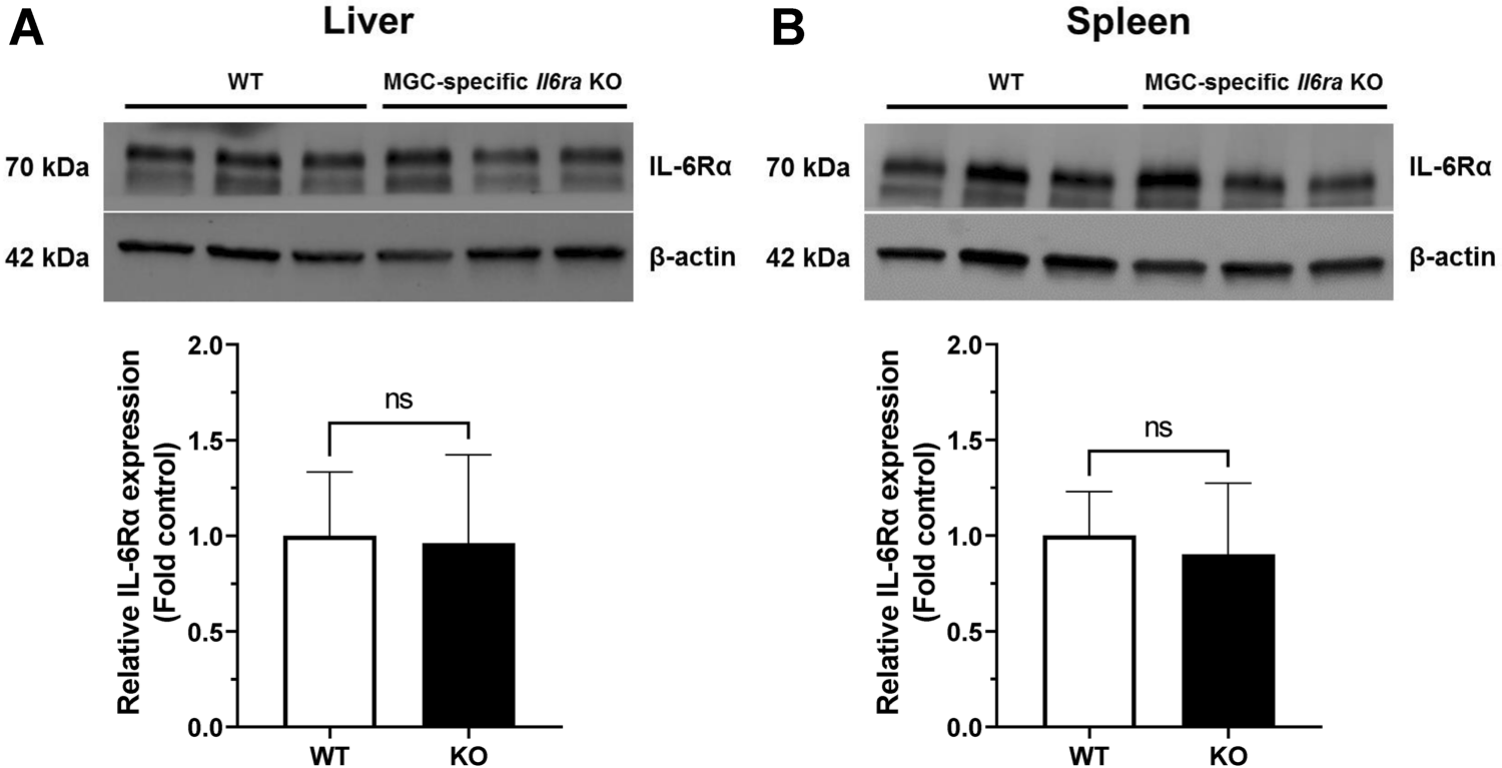
Müller-glial-cell-specific *Il6ra*^*−/−*^ mice retain IL-6Rα expression in the liver and spleen. The specificity of IL-6Rα knockout to Müller glial cells was confirmed by western blot analysis of homogenates from tissues known to express IL-6Rα. (**A**) Liver and (**B**) spleen from controls and MGC-specific *Il6ra* KO mice were probed for the IL-6 receptor (70 kDa) and normalized to β-actin (42 kDa). Data is expressed as fold control mean ± SD, n = 4–5/group, WT (littermate controls: *wt/fl* or *fl/fl*); ns, not significant (p-value ≥ 0.05 vs. WT, unpaired T-test).

To evaluate the specificity of *Il6ra* knockout to Müller glia, we confirmed that expression of IL-6Rα was maintained in hepatocytes and leukocytes, cell types that express the membrane-bound IL-6 receptor under healthy physiologic conditions^48,49^, using tissue homogenates isolated from liver and spleen. After normalizing to β-actin, liver homogenates of the knockout strain retained IL-6Rα with no significant change in expression relative to littermate controls (*wt/fl* or *fl/fl* mice) without *Cre* expression (FC = 0.961, n = 4–5/group; p-value = 0.8856 vs. control, unpaired T-test) (Fig. 5A). Similarly, the expression of IL-6Rα in the spleen was not significantly changed in MGC-specific *Il6ra* knockout mice (FC = 0.903, n = 4–5/group; p-value = 0.6438 vs. control, not significant, unpaired T-test) (Fig. 5B). Despite biological variability between animals in each group, knockout mice retained IL-6Rα expression in these organs, indicating that systemic functionality of IL-6 signaling remains intact in tissues and cells outside of Müller glia.

### Müller-glial-cell-specific ***Il6ra***^***−/−***^ mice retain retinal morphology and function similar to wildtype mice

**Figure 6:**
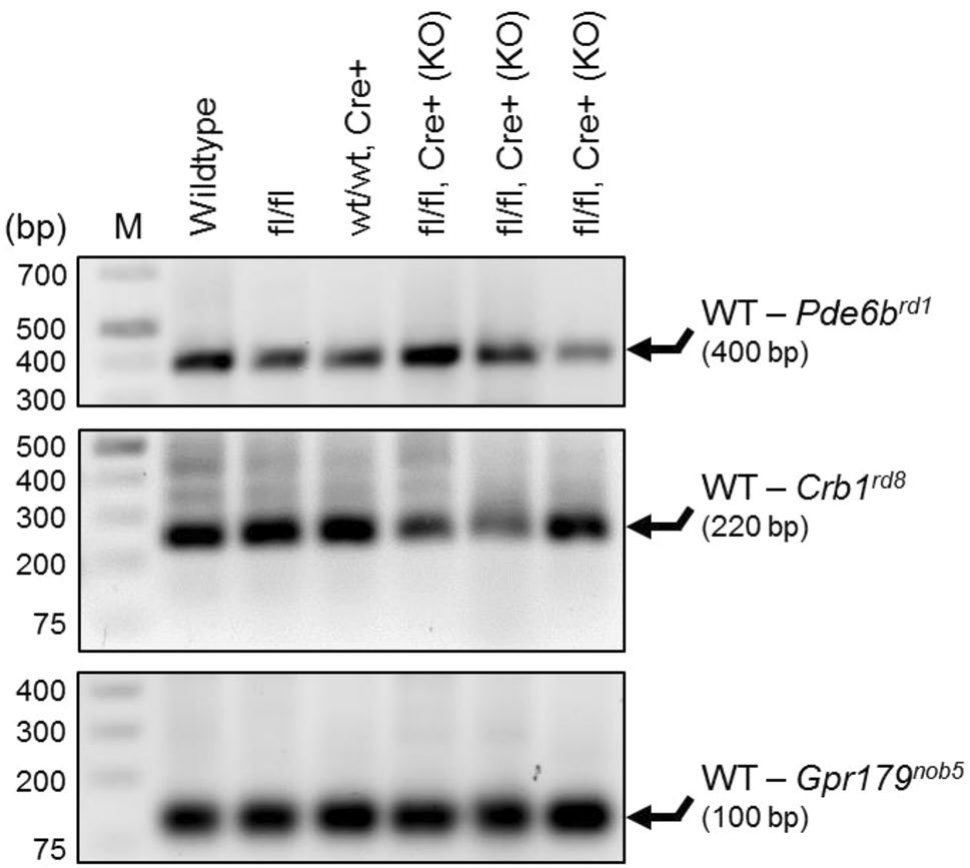
Genetic mutations associated with retinal degeneration phenotypes are not present in Müller-glial-cell-specific *Il6ra*^*−/−*^ mice. Parental mice of the MGC-specific *Il6ra* KO (male B6;SJL-*Il6ra*^*tm1.1Drew*^/J and female C57BL/6-Tg(Pdgfra-cre)1Clc/J) and KO offspring were screened for known genetic mutations associated with the background of C57BL/6J or /6NJ mice—*Pde6b* (*rd1*), *Crb1* (*rd8*), and *Gpr179* (*nob5*)—by allele-specific PCR of isolated genomic DNA. Representative agarose gel for PCR products from wildtype, F0 *Il6ra* floxed (fl/fl), F0 *Pdgfra-Cre* (wt/wt, Cre+), and F2-F3 MGC-specific *Il6ra*^*−/−*^ mice (fl/fl, Cre+) are shown. n = 1–3/group; M, GeneRuler 1 kb Plus DNA Ladder.

The Müller-glial-cell-specific Cre recombinase strain (C57BL/6-Tg(Pdgfra-cre)1Clc/J) was originally generated in a C57BL/6 mouse with a mixed background consisting of C57BL/6J and C57BL/6N substrains. The C57BL/6N substrain has been shown to carry the *rd8* mutation in the *Crb1* gene, which results in retinal degeneration and characteristic ocular lesions^45,50^. Because of this, the *Pdgfra-Cre* female mice were screened alongside the *Il6ra*-floxed male mice (B6;SJL-*Il6ra*^*tm1.1Drew*^/J) for genetic mutations known to cause retinal degeneration—*Crb1* (*rd8*), *Pde6b* (*rd1*), and *Gpr179* (*nob5*)^45,50–54^. Genomic DNA from these F0 parental strains and MGC-specific *Il6ra*^*−/−*^ mice was compared to wildtype (C57BL/6J) mice using allele-specific PCR as previously described^45,50^. Agarose gel electrophoresis of PCR products revealed that only wildtype bands for each primer set were present in all strains and generations (Fig. 6). For *Pde6b*, one band was visible at 400 bp, while the *rd1* mutant band at 550 bp was not observed. A prominent band for the wildtype *Crb1* allele was present at 220 bp, whereas no other bands were observed at the *rd8* mutant length of 244 bp. The wildtype band for *Gpr179* was visible at 100 bp for all genotypes tested, and no bands were observed to indicate the presence of the *nob5* mutant at 400 bp in the mice used in this study. Thus, neither of the parental strains nor the knockout offspring carry any of the mutations tested, indicating that MGC-specific *Il6ra*^*−/−*^ mice are not genetically predisposed to or carriers of these mutations commonly associated with retinal degeneration phenotypes in mice.

**Figure 7:**
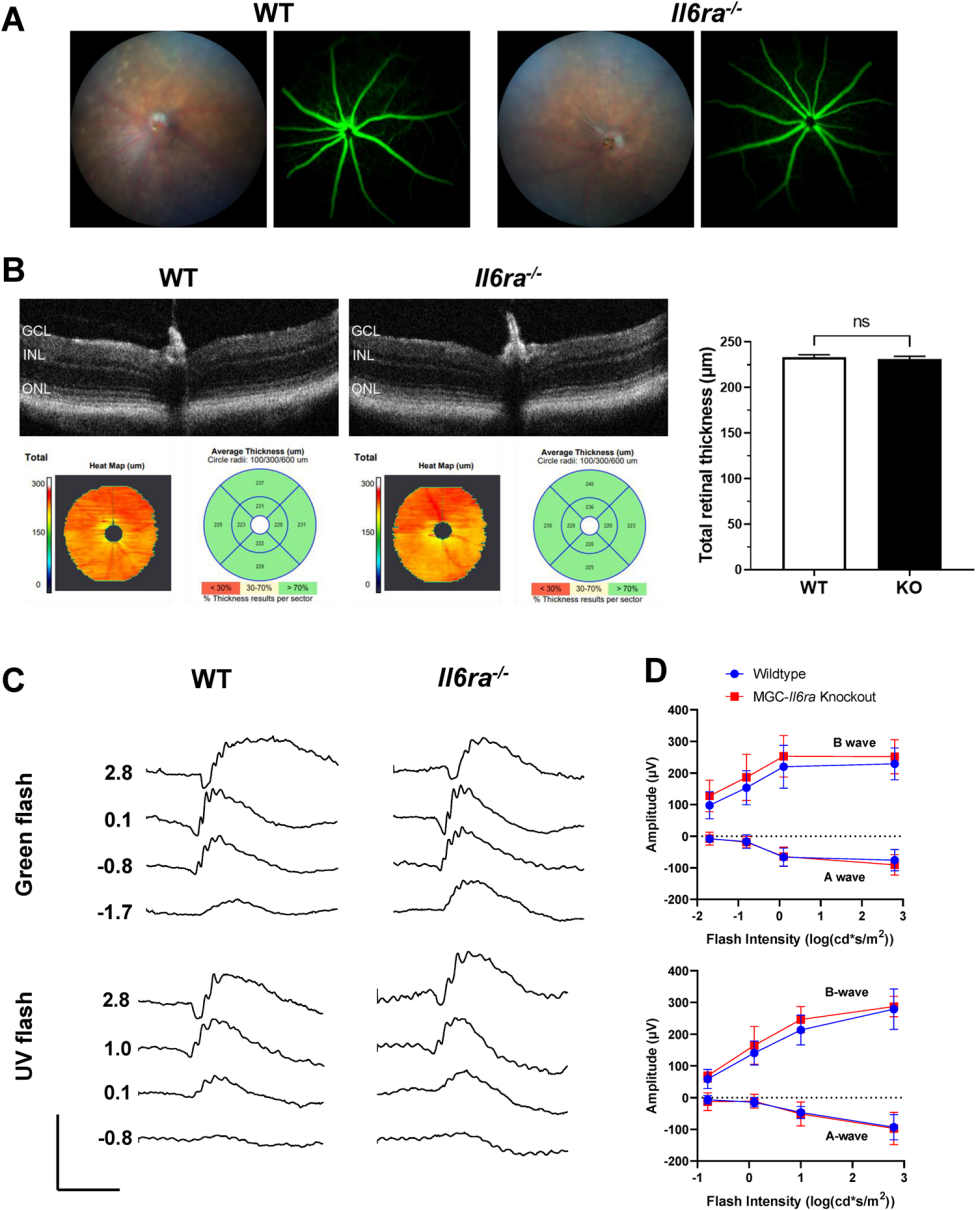
Müller-glial-cell-specific *Il6ra*^*−/−*^ mice retain the retinal morphology and the ERG response of wildtype C57BL/6J mice in vivo. (**A**) Representative fundoscopy and fluorescein angiography images for KO and WT mice (right eye). (**B**) Average total retinal thickness measurements in vivo as measured by optical coherence tomography (OCT) of MGC-specific *Il6ra*^*−/−*^ mice and WT mice. Data shows representative OCT scans for WT and KO mice; bar graph shows mean ± SD, n = 5–6 animals/group (right eyes; 5–6 eyes/group; 22–28 weeks old); ns, not significant (p-value = 0.3325 *vs.* WT, unpaired T-test). (**C**) Representative electroretinography (ERG) waveforms of dark-adapted KO and littermate control (WT) mice over increasing flash intensities of green and UV light (log(cd s/m^2^)). (**D**) The A-wave and B-wave amplitudes (µV) measured by ERG were compared at each flash intensity. Data is represented as mean ± SD, n = 8–9 animals/group (16–18 eyes/group); wildtype (WT, n = 9 animals, 18 eyes; 12–40 weeks old), *Il6ra*^*−/−*^ (KO, n = 8 animals, 16 eyes; 12–38 weeks old); ns, not significant (p-value ≥ 0.05 vs. WT, unpaired T-test of each flash intensity).

Preliminary analyses of the retinal morphology and the retinal responses of Müller-glial-cell-specific *Il6ra*^*−/−*^ mice were performed as a baseline characterization of the strain relative to littermate controls and wildtype (C57BL/6J) mice for future studies (Fig. 7). Live retinal imaging of knockout mice revealed no visible lesions or abnormalities on fundus examination, and no significant alterations in the retinal vasculature were observed following fluorescein angiography in wildtype and knockout mice (Fig. 7A). Average total retinal thickness was measured by optical coherence tomography (OCT) in MGC-specific *Il6ra*^*−/−*^ mice and wildtype mice between 22 to 28 weeks of age (Fig. 7B). Measurements from each group revealed a total average thickness of 230.91 µm for knockout and 232.88 µm for wildtype, revealing no significant difference in total retinal thickness after MGC-specific loss of membrane-bound IL-6Rα. Electroretinography (ERG, Ganzfeld) of dark-adapted knockout and littermate control mice (12–40 weeks old) was also performed to assess the retinal responses of rods and S-cones over increasing flash intensities of green and UV light (log(cd s/m^2^)), respectively, in MGC-specific *Il6ra*^*−/−*^ mice (Fig. 7C). Measurements were obtained from both left and right eyes of each mouse following overnight dark adaptation for each group. A-wave and B-wave amplitudes (µV) were compared at each flash intensity to identify any baseline differences in the retinal function of the knockout strain. After comparing each flash intensity between the two groups, no significant changes were observed for either rod function (green light stimulus) or S-cones (UV light stimulus) between groups (Fig. 7D). This data indicates a similar ERG response in MGC-specific *Il6ra*^*−/−*^ mice and their littermate controls (*wt/fl*, *fl/fl,* and *wt/fl Cre* +) despite the cell-specific loss of membrane-bound IL-6Rα in the knockout.

## Discussion

Müller glial cells (MGCs) play an important role in retinal function including the maintenance of the blood-retinal barrier and the induction of inflammatory response in diabetic retinopathy (DR). Increased IL-6 levels are a hallmark of DR and function through two pathways. In this study, we successfully generated and characterized a Müller-glial-cell-specific IL-6 receptor knockout mouse strain. This novel tissue-specific knockout mouse will allow for the delineation of the specific effects of IL-6 trans-signaling within Müller glial cells in vivo without the activation of classical signaling. This will help in differentiation of the two signaling mechanisms within MGCs. This strain was generated using Cre-*loxP* recombination by crossing two existing strains, C57BL/6-Tg(Pdgfra-cre)1Clc/J and B6;SJL-*Il6ra*^*tm1.1Drew*^/J. C57BL/6-Tg(Pdgfra-cre)1Clc/J, originally developed by the Cepko laboratory^36^, expresses Cre in the cell bodies of the inner nuclear layer (INL) of the retina, but some expression may be observed in the outer nuclear layer (ONL) and in the ganglion cell layer (GCL)^35^. The B6;SJL-*Il6ra*^*tm1.1Drew*^/J strain was developed by the Drew laboratory to study the specific effects of IL-6R and IL-6 classical signaling in wound healing^37^. Crossing these strains resulted in a successful Müller-glial-cell-specific knockout of IL-6Rα in the mouse retina.

Our studies show successful deletion of functional IL-6 receptor expression in MGCs, both within retinal sections and in Müller glial cells isolated from these knockout mice. Normal IL-6Rα expression was observed in the liver and spleen, confirming that the knockout was tissue-specific. Furthermore, this strain showed no gross or obvious histologic changes on funduscopic exam or in retinal tissue sectioning. The knockout mice showed no changes in response to visual stimuli compared to wildtype as measured by ERG. These studies confirm successful generation of an MGC-specific “IL-6 trans-signaling only” mouse model, in which the specific effects of IL-6 trans-signaling in MGCs can be studied in vivo or in isolated cells in vitro without any confounding effects of classical signaling.

In future studies a “trans-signaling only” strain could be used alongside wildtype mice to indirectly measure the effects of classical signaling in MGCs. Furthermore, these mice could also be studied with existing “classical signaling only” strains, which constitutively express the trans-signaling inhibitor sgp130Fc in the liver^55^ or in the central nervous system^56^, to thoroughly delineate the specific activities of classical and trans-signaling in MGCs both directly and indirectly. Additionally, Müller glial cells isolated from this strain survive and proliferate in culture like wildtype MGCs, allowing for the effective study of IL-6 trans-signaling in MGCs both in vivo and in vitro*.*

Our primary interest in IL-6 trans-signaling is in the context of the development and progression of diabetic retinopathy. To evaluate the effect of trans-signaling without classical signaling activation in this context, diabetes can be induced in this strain using streptozotocin (STZ), a well-established method to induce a diabetic phenotype through toxin-induced destruction of pancreatic beta-cells^57^. Using this model, trans-signaling in the absence of classical signaling can be studied longitudinally throughout the course of disease development beginning with a pre-diabetic state. This model also has potential uses in other retinal diseases. For example, IL-6 has been implicated in the development of retinopathy of prematurity and other ischemic retinopathies, and use of an oxygen-induced retinopathy model with this Müller-glial-cell-specific *Il6ra*^*−/−*^ strain could delineate the specific functions of IL-6 classical and trans-signaling in these retinal diseases^58–62^.

In conclusion, we have successfully generated a novel Müller cell-specific *Il6ra*^*−/−*^ mouse strain using Cre-*loxP* recombination and have confirmed knockout of IL-6Rα expression in MGCs in retinal tissue sections and isolated MGCs. This new model will allow us to isolate the specific effects of IL-6 trans-signaling in Müller glial cells and differentiate the roles of classical and trans-signaling in retinal disease. Use of this model will provide critical knowledge of the often conflicting effects of the two major IL-6 signaling modalities in retinal pathology, opening the door for more effective and targeted anti-inflammatory therapies for ocular disease.

## Supplementary Information

Supplementary Information.

## Data Availability

All the data used in the current study are available either in the main text or as Supplemental Material.
