# Supplementary material for: Generation and characterization of a Müller-glial-cell-specific Il6ra knockout mouse to delineate the effects of IL-6 trans-signaling in the retina
Source: Sci Rep. 2022 Oct 21;12:17626. doi: 10.1038/s41598-022-22329-3 (PMC9587029; doi:10.1038/s41598-022-22329-3)
Supplement: Supplementary file 1 — Supplementary Information. [file 41598_2022_22329_MOESM1_ESM.pdf]

Supplemental Table 1. Genotyping and allele-specific primers.

Gene	Forward Sequence (5'→3')	Reverse Sequence (5'→3')	Product length
Cre	GAGTGATGAGGTTCGCAAGA	CTACACCAGAGACGGAAATC	635 bp
IL6ra WT	CTGCCTCCATCTCCCAAGT	GCACCTTGACCCTACACACA	109 bp
IL6ra fl/fl	CTGCCTCCATCTCCCAAGT	TCCTATTCCGAAGTTCCTATTCTC	123 bp
Pde6b WT	TACCCACCCTTCCTAATTTTTCTCAGC	TGACAATTACTCCTTTTCCCTCAGTCTG	400 bp
Pde6b Mut	GTAAACAGCAAGAGGCTTTATTGGGAAC		550 bp
Crb1 F1	GTGAAGACAGCTACAGTTCTGATC	GCCCCATTTGCACACTGATGAC	220 bp
Crb1 F2 Mut	GCCCCTGTTTGCATGGAGGAAACTTGGAAG ACAGCTACAGTTCTTCTG		244 bp
Gpr179 WT	TGTGCCTGGGTATCTGTTGA	GCTTACACACTTACACACAGATAGAT	100 bp
Gpr179 Mut	GCATGTGCCAAGGGTATCTT		400 bp

Supplemental Table 2. Primer sets for quantitative RT-PCR analysis of isolated MGCs.

Gene	Forward Sequence (5'→3')	Reverse Sequence (5'→3')
Il6ra E2-E3	GGCCACCGTTACCCTGATTT	GCTCCTCTGGGGGAACATCC
Il6ra E5-E6	AAGGAGTTCACGGTGTTGCT	GTGGTCCTGGGCTCTATCCA
Il6ra E10	TGAATGATGACCCCAGGCAC	ACACCCATCCGCTCTCTACT
Cre	CGACCAGGTTCGTTCACTCA	CAGCGTTTTCGTTCTGCCAA
Gfap	CCTCCAGATCCGAGAAACCAG	TTGTGCTCCTGCTTCGAGTC
Gapdh	CCCTTAAGAGGGATGCTGCC	TACGGCCAAATCCGTTCACA

Supplemental Figure 1.

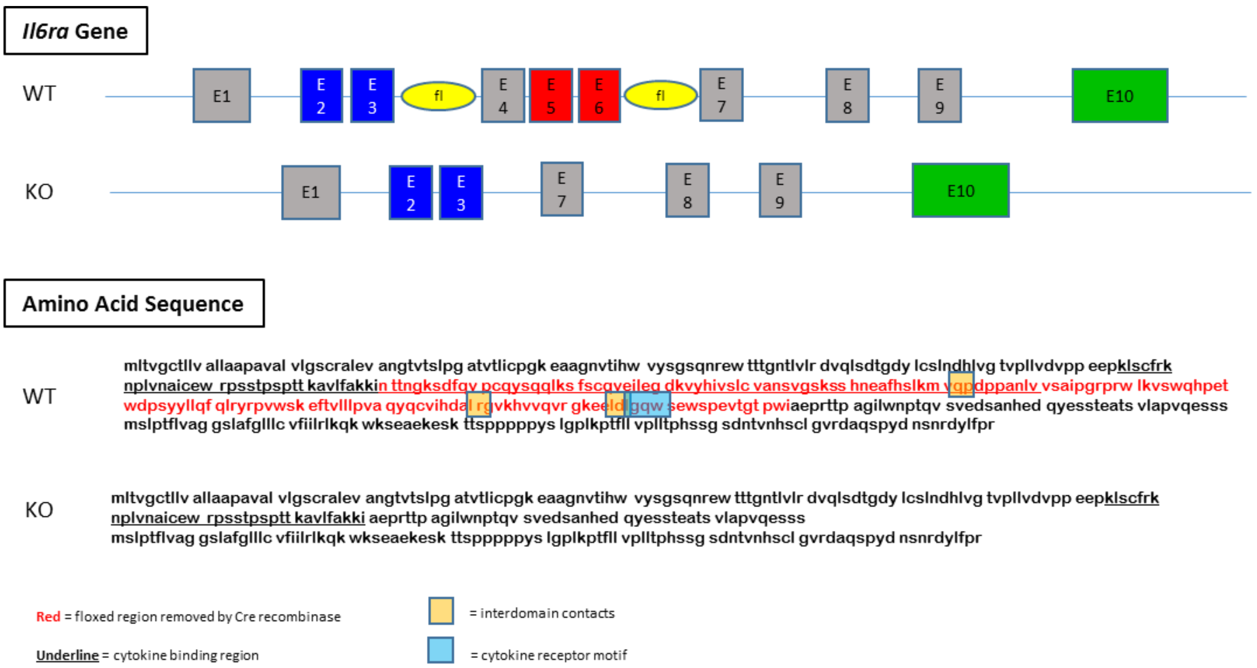

Supplemental Figure 2.

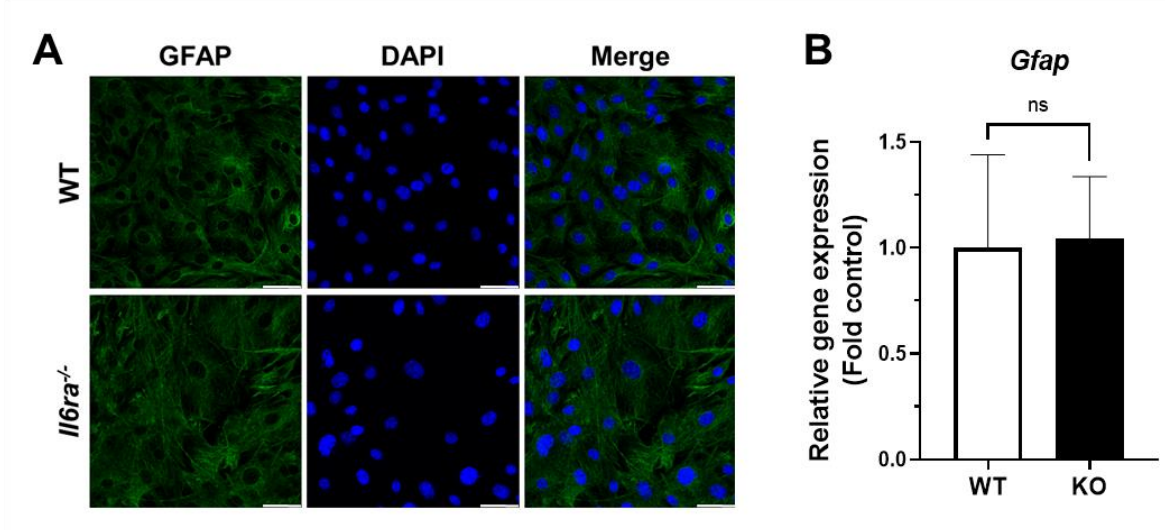

(A) Representative immunofluorescence and (B) RT-PCR analysis of *Gfap* expression in KO MGCs shows no evidence for increased glial cell activation. GFAP (A0237, anti-rabbit, 1:100, green (Alexa-Fluor 488, 1:500)), DAPI (1:2000, blue), n=6/group; WT: wildtype MGCs; *Il6ra*$^{-/-}$: knockout MGCs. Bar graphs are represented as means ± SD, n=6/group; ns, not significant (p-value = 0.8453; unpaired T-test, KO *vs.* WT).
